# Neuro-toxoplasmosis in haploidentical haematopoietic stem cell transplant

**DOI:** 10.1007/s00277-025-06281-6

**Published:** 2025-02-26

**Authors:** Marco Galli, Lorenzo Masina, Gabriele Magliano, Enrico Morello, Evelyn Van Hauwermeiren, Michele Malagola, Mirko Farina, Vera Radici, Domenico Russo, Daniele Avenoso

**Affiliations:** 1 Unit of Blood Diseases and Bone Marrow Transplantation, Department of Clinical and Experimental Science, University of Brescia, ASST Spedali Civili di Brescia, Brescia, Italy; 2 Division of Infectious and Tropical Disease, ASST Spedali Civili Hospital, Brescia, Italy

**Keywords:** Toxoplasmosis, haploidentical stem cell transplant, PTCY, MDS

## Abstract

Allogeneic haematopoietic stem cell transplant is a routine procedure for several haematological disorders though infections are the main cause of morbidity and mortality. Rare infections, such as protozoan reactivations, can be life-threatening and clinicians should be aware of these possibilities. Herein, we present a case of neuro-toxoplasmosis post haploidentical haematopoietic stem cell transplant.

Allogeneic haematopoietic stem cell transplant (alloHSCT) is a curative treatment for several haematological disorders, though infections remain a significant risk of morbidity. Most infections are sustained by bacteria, virus and fungi, and rarely by protozoan; indeed, protozoan infections occur in between 0.31% and 10% of alloHSCT patients [1–2]. *Toxoplasma gondii*, an intracellular protozoan parasite, can cause tissue infection, mainly within the eyes and central nervous system. Its incidence varies, with higher rates in countries with high toxoplasmosis prevalence in the general population (0.2-5.7%) [3]. Immunocompetent subjects usually have asymptomatic infection but immunocompromised patients can develop *Toxoplasma* disease due to its reactivation. [4–5]. Neurotoxoplasmosis in alloHSCT patients presents within the first 2 months of transplant. Because of non-specific symptoms, its diagnosis is difficult and it becomes evident at post-mortem examination [6]. Diagnosis is based on the detection of *Toxoplasma* DNA through positive PCR [7]. Combining clinical suspicion, imaging, and laboratory study is fundamental to achieve the diagnosis. Prophylaxis with trimethoprim-sulfamethoxazole (TMP/SMZ) can prevent this complication [5], but in early phase of alloHSCT TMP/SMZ may not be feasible due its known myelotoxic profile [8]. The lack of prophylaxis is the main reason for observing a higher incidence of this complication in the early phase of transplant [9].

Herein we present the case of a 75-year-old Toxoplasma IgG positive patient with myelodysplastic syndrome (MDS) who underwent reduced intensity haploidentical HSCT. Graft-*versus-*host disease prophylaxis was with post-transplant cyclophosphamide, mycophenolate and tacrolimus, as previously reported [10]. Neutrophil and platelet engraftment occurred on day + 24 and + 22, respectively. Although there was no evidence of peripheral consumption, the platelet counts consistently remained below 40,000/mm³, and neutrophil levels fluctuated between 1,100 and 1,500/mm³. These findings were interpreted as indicative of poor graft function, leading to a delay in initiating the standard prophylaxis with TMP/SMZ. On day + 40 the patient developed low grade fever and speech disturbances, confusion, somnolence, depression, memory deficits, muscle tremors, spatial and temporal disorientation. The electroencephalogram evidenced a global slow wave activity without sign of focal lesions. Magnetic resonance imaging (MRI) of the central nervous system (CNS) showed small lesion (7 × 11 mm) within the subcortical white matter of the left cerebellar hemisphere, characterized by vasogenic oedema and ring enhancement without causing intracranial mass effect on the brainstem and fourth ventricle, suggestive for abscess. A diagnostic lumbar puncture was performed to rule out MDS localisation or viral infection. Polymerase chain reaction (PCR) on cerebro-spinal fluid detected *Toxoplasma DNA*, thus treatment with high dose TMP/SMZ (3600/720 mg/die for six weeks) was started. Morphology evaluation of CSF allowed to rule out MDS localisation within the CNS. Three weeks after TMP/SMZ therapy, follow up CNS MRI showed initial reduction in the size of the cerebellar lesion (6 × 10 mm) and of the vasogenic oedema without new lesions. Concomitantly, there was progressive improvement of the neurological symptoms. Six weeks after the diagnosis of neuro-toxoplasmosis there was complete resolution of the neurological symptoms without lifelong sequelae.

This case underscores the importance of a complete diagnostic work up, where the integration between clinical evaluation, baseline serologies, radiological investigations and molecular assay of CSF allowed the diagnosis of a rare opportunistic infection. The multidisciplinary approach allowed the prompt initiation of TMP/SMZ that is likely the reason for the lack of lifelong neurological sequelae. In conclusion, albeit neuro-toxoplasmosis is still a rare complication, all Toxoplasma IgG positive alloHSCT patient with neurological symptoms should undergo diagnostic lumbar puncture.

## Data Availability

No datasets were generated or analysed during the current study.
